# A systematic review and meta-analysis of the efficacy and safety of mirabegron vs. tamsulosin in alleviating double-J catheter related symptoms

**DOI:** 10.3389/fmed.2025.1669827

**Published:** 2025-11-03

**Authors:** Jiankun Zhang, Dawei Wang, Junpeng Chi, Keyuan Lou, Jinsheng Wu, Yuanshan Cui

**Affiliations:** ^1^School of Clinical Medicine, Shandong Second Medical University, Weifang, China; ^2^Department of Urology, Yantai Yuhuangding Hospital, Yantai, China; ^3^Department of Urology, Weifang People's Hospital, Weifang, China; ^4^Department of Urology, Affiliated Hospital of Shandong Second Medical University, Weifang, China

**Keywords:** mirabegron, tamsulosin, double J stent, safety, efficacy, meta-analysis

## Abstract

**Background:**

Currently, many clinicians use tamsulosin as a medication to alleviate symptoms following double J (DJ) stent insertion. With the emerging advantages of *β*-receptor agonists in relieving urinary tract spasms, the effectiveness of mirabegron in treating DJ stent-related symptoms has gained attention.

**Objective:**

To explore which medication, mirabegron or tamsulosin, is more effective in alleviating DJ stent-related symptoms.

**Methods:**

A literature search was conducted to identify randomized controlled trials (RCTs) that evaluated the use of mirabegron and tamsulosin in relieving DJ stent-related symptoms. Databases searched included PubMed, Web of Science, Cochrane Library, Embase, and CNKI, with the search cutoff date in June 2024. Inclusion criteria were patients who underwent percutaneous nephrolithotomy (PCNL) or ureteroscopy (URS) with DJ stent placement and were treated with either tamsulosin or mirabegron. Data extraction and quality assessment were performed using standardized methods, and a meta-analysis was conducted.

**Results:**

One of the Ureteral Stent Symptom Questionnaire (USSQ) indicators, Work Performance Index (WPI), showed a mean difference (MD) of −1.01 (95% CI: −1.91 to −0.11, *p* = 0.03, *I*^2^ = 77%). For side effects: risk ratios (RR) = 0.34, (95% CI = 0.13 to 0.89, *p* = 0.03, *I*^2^ = 0%).

**Conclusion:**

This study shows that mirabegron demonstrates better efficacy in terms of WPI and has fewer side effects overall, particularly for patients experiencing tamsulosin-related side effects such as hypotension or ejaculatory dysfunction. However, no significant differences were found between the two drugs in other aspects of the USSQ or in the IPSS. Further large-scale, high-quality RCTs with longer follow-up periods and comprehensive safety data are needed to confirm these findings and identify patient groups who may benefit most from mirabegron treatment.

**Systematic review registration:**

https://www.crd.york.ac.uk/prospero/, CRD420251083374.

## Introduction

Percutaneous nephrolithotomy (PCNL) and ureteroscopy (URS) are common surgical methods for the treatment of kidney stones and ureteral stones (1). Postoperatively, surgeons typically place a ureteral stent to facilitate stone passage and reduce the occurrence of postoperative complications (2). However, the use of double J (DJ) stents may lead to a range of stent-related symptoms (3), such as hematuria, pain, urinary tract infections, and bladder irritability. Studies have shown that oral medications can effectively alleviate these symptoms (4).

Alpha-blockers and anticholinergic drugs have been shown to be effective in improving stent-related symptoms (SRS) (5). Among them, tamsulosin, an *α*-blocker, has been widely used in clinical treatment. Mirabegron, the first β3-receptor agonist, has demonstrated excellent efficacy in overactive bladder (6). Recent studies suggest that mirabegron also shows promising efficacy in managing DJ stent-related symptoms (7). Joshi et al. developed and validated the Ureteral Stent Symptom Questionnaire (USSQ) to assess DJ stent-related symptoms (8), which has been widely used for evaluating these symptoms.

Randomized controlled trials (RCTs) have explored the differences in efficacy between mirabegron and tamsulosin, but the results are inconsistent. This study aims to conduct a meta-analysis using the USSQ scoring system to perform a head-to-head comparison of the efficacy of mirabegron and tamsulosin, providing clearer clinical guidance.

## Methods

This meta-analysis was reported in accordance with the PRISMA (Preferred Reporting Items for Systematic Reviews and Meta-Analyses) guidelines and has been prospectively registered on the International Prospective Register of Systematic Reviews (registration number: CRD420251083374).

### Search strategy

We searched PubMed, Web of Science, Embase, Cochrane Library, China National Knowledge Infrastructure (CNKI), and ResearchGate. The search cutoff date was June 1, 2025, with no language restrictions. The search terms included “mirabegron,” “tamsulosin,” “randomized controlled trial,” and “Double J Stent.” The search results included 14 articles from the Cochrane Library, 55 articles from PubMed, 15 articles from Web of Science, 104 articles from Embase, 7 articles from CNKI, and 4 articles from ResearchGate.

### Inclusion and exclusion criteria

Strict inclusion and exclusion criteria were applied. We selected RCTs comparing the efficacy of mirabegron and tamsulosin, involving patients aged 18 years or older who underwent PCNL or URS followed by double J stent placement and reported outcomes using the USSQ or International Prostate Symptom Score (IPSS). The exclusion criteria were: observational studies, retrospective studies, studies with an intervention period of less than 2 weeks, and studies that did not evaluate the efficacy of mirabegron and tamsulosin. Non-comparative studies, studies with no outcome reports, studies with missing data, as well as reviews and meta-analyses, were also excluded.

Two independent reviewers screened the titles and abstracts of the studies and retrieved the full-text studies that met the inclusion criteria. Studies selected for in-depth analysis were reviewed by both researchers, and disagreements were resolved by a third reviewer.

### Data extraction

Data including authors, year, sample size, distribution, study population, country of the trial, follow-up duration, primary outcome measures (USSQ), and secondary outcome measures (VAS, IPSS) were independently extracted into tables by two reviewers. Any discrepancies were resolved through discussion (Table 1 and Supplementary Table 1).

**Table 1:** The characteristics of the included studies

Number	First author	Duration of the study	Year	Country of publication	Study design	Population characteristics	Sample size	Arms	Intervention
1	Chandna A, Kumar S	2018.10–2020.1	2021	India	Randomized Controlled Trial	>18 patients; Ureteral stent placement	83	Mirabegron (*n* = 41) Tamsulosin (*n* = 42)	M OR T (4 weeks)
2	Yavuz A, Kilinc MF	2017.2–2018.5	2020	Turkey	Randomized Controlled Trial	>18 patients; Ureteral stent placement	105	Mirabegron(*n* = 50) Tamsulosin(*n* = 55)	M OR T (4 weeks)
3	Javid M, Abdullah A	2021.1–2021.10	2023	India	Randomized Controlled Trial	>18 patients; Ureteral stent placement	80	Mirabegron(*n* = 40)Tamsulosin(*n* = 40)	M OR T (15 days)
4	King Alexander, Achmad M.	2019.7–2019.11	2020	Indonesia	Randomized Controlled Trial	>18 patients; Ureteral stent placement	50	Mirabegron(*n* = 25)Tamsulosin(*n* = 25)	M OR T (3 weeks)
5	Hafiz Muhammad Yousaf Khan	2019.5–2019.11	2022	Pakistan	Randomized Controlled Trial	>18 patients; Ureteral stent placement	100	Mirabegron(*n* = 50)Tamsulosin(*n* = 50)	M OR T (3 weeks)
6	Manish Garg	2016.1–2018.2	2022	India	Randomized controlled trial	>18 patients; Ureteral stent placement	70	Mirabegron(*n* = 35)Tamsulosin(*n* = 35)	M OR T (2 weeks)
7	Cătălin Pricop, Carina Alexandra Bandac	2022.1–2023.8	2024	Romania	Randomized Controlled Trial	>18 patients; Ureteral stent placement	106	Mirabegron(*n* = 51)Tamsulosin(*n* = 55)	M OR T (30 days)
8	Akrm A. Elmarakbi	2020.6-	2024	Egypt	Randomized Controlled Trial	>18 patients; Ureteral stent placement	100	Mirabegron(*n* = 50)Tamsulosin(*n* = 50)	M OR T (2 weeks)
9	Mao Min	2021.6–2022.11	2023	China	Randomized Controlled Trial	>18 patients; Ureteral stent placement	130	Mirabegron(*n* = 65)Tamsulosin(*n* = 65)	M OR T (2–4 weeks)

### Quality assessment

The risk of bias for each study was assessed using the Cochrane Risk of Bias tool for RCTs. Bias was categorized as selection bias, performance bias, detection bias, attrition bias, reporting bias, and other bias, with each being classified as low, high, or unclear risk. Two independent reviewers assessed the risk of bias according to PRISMA guidelines.

### Statistical analysis

RevMan was used to compare mirabegron and tamsulosin, analyzing continuous data such as USSQ, VAS, and IPSS, and calculating mean differences (MD) with 95% confidence intervals (CI). For dichotomous outcomes (side effects), risk ratios (RR) and 95% CI were computed. In cases where standard deviation (SD) or standard error (SE) was not reported, missing data were estimated using other available statistical information (such as mean and standard error) or directly obtained from the authors when necessary.

By analyzing the different dimensions of the USSQ, we were able to assess the differential effects of mirabegron and tamsulosin on various SRS. This method allowed us to gain deeper insight into how each drug targets specific symptoms. IPSS and VAS were used as secondary outcome measures to evaluate the effects of treatment on urinary tract irritation. To assess heterogeneity across studies, the I^2^ statistic was used. The *I*^2^ value represents the proportion of variance in the effect size due to real differences rather than random error. When the *I*^2^ value was below 50%, a fixed-effect model was used, assuming homogeneous effect sizes; when the *I*^2^ value exceeded 50%, a random-effect model was employed to account for differences between studies. To assess the sources of heterogeneity, we conducted a sensitivity analysis by sequentially removing each study and recalculating the pooled effect and heterogeneity indices.

Funnel plots were used to assess publication bias. Symmetrical funnel plots indicated no significant bias, while asymmetrical funnel plots, especially if small-scale studies clustered on one side, might indicate publication bias. Statistical significance was assessed using *p*-values (<0.05). A *p*-value less than 0.05 was considered statistically significant, indicating that the observed results were unlikely to have occurred due to random error.

## Results

### Characteristics of the individual studies

**Figure 1:**
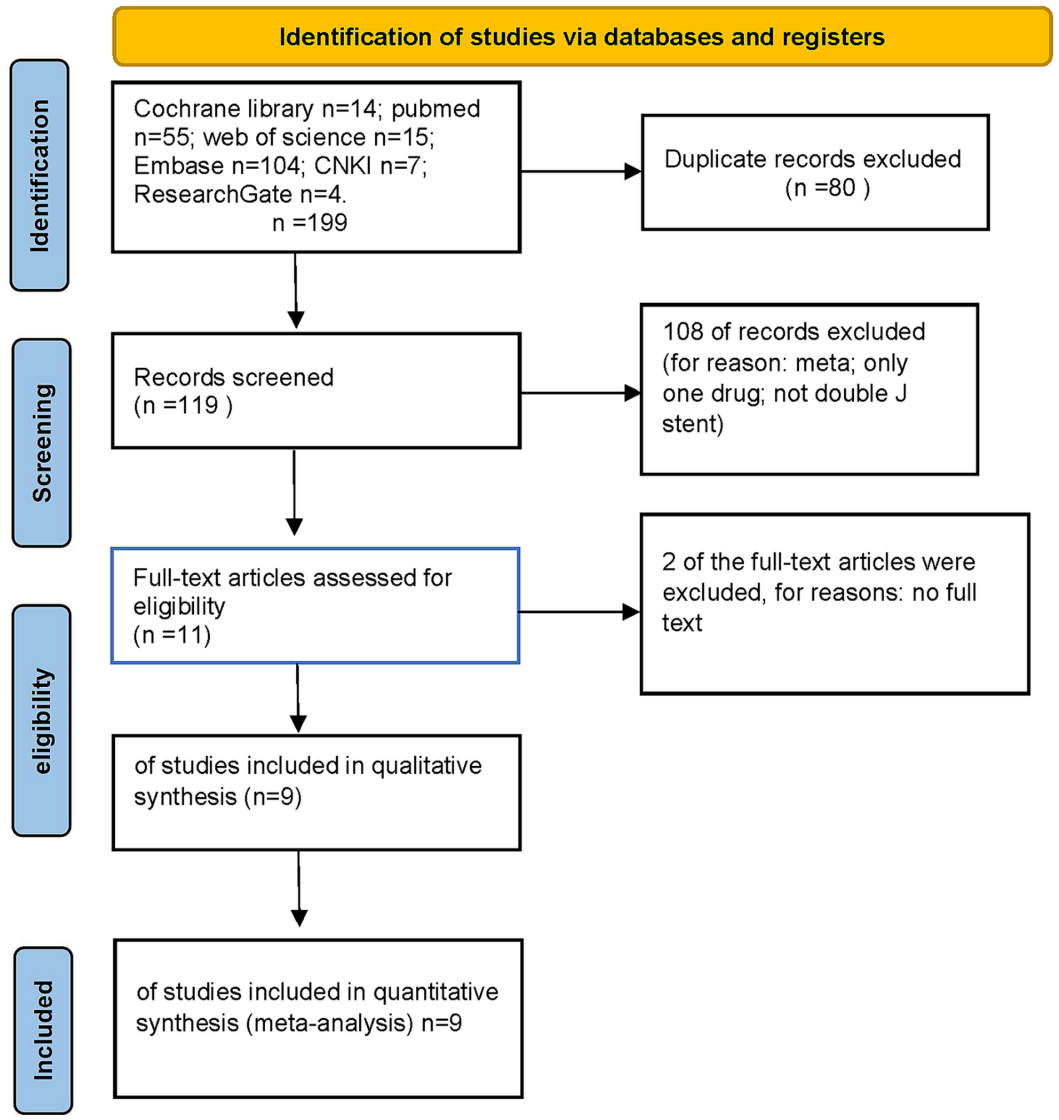
PRISMA flow diagram.

Through database searching, a total of 195 potentially eligible articles for inclusion in our meta-analysis were identified. Based on the inclusion and exclusion criteria, 186 articles were excluded after screening titles and abstracts. Ultimately, 9 studies met the inclusion criteria and were included in the meta-analysis (9–16, 34). The selection process and reasons for exclusion are detailed in the flow diagram (Figure 1). All included studies provided relevant information for USSQ or IPSS, and four studies described changes in VAS after treatment.

### Quality of individual studies

**Figure 2:**
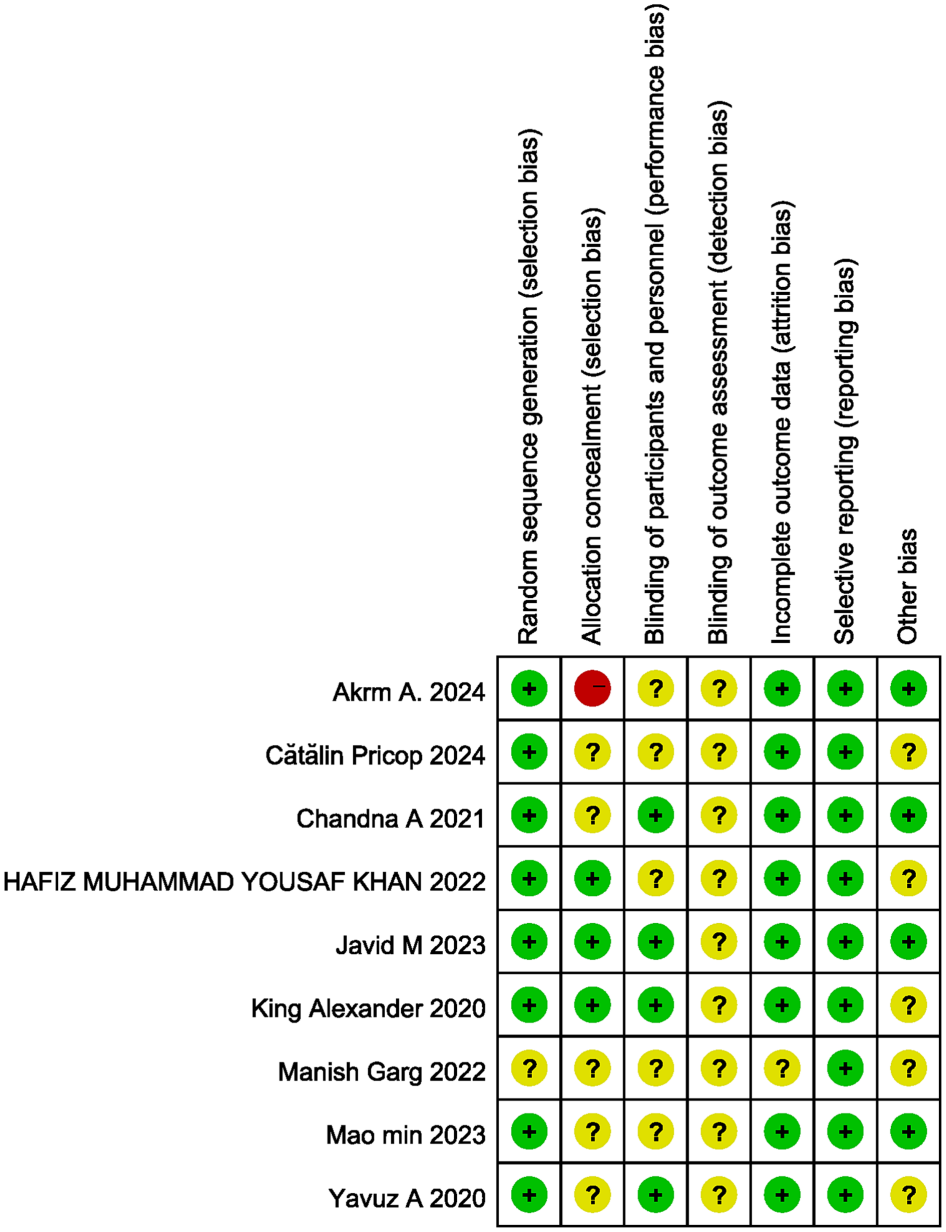
Quality assessment of individual study.

All 9 studies were RCTs and described their randomization process. Each study performed a power calculation for the sample size. According to the Cochrane Library’s revised RCT quality assessment tool, all included studies were classified as “low risk of bias” (Figure 2). The funnel plot qualitatively assessed publication bias in the included studies (Supplementary Figure 1). The plot is symmetric, indicating no evidence of publication bias, suggesting that publication bias was not a significant issue in the included studies. No formal statistical test (e.g., Egger’s test) was performed to assess the asymmetry of the funnel plot.

### USSQ and VAS

**Figure 3:**
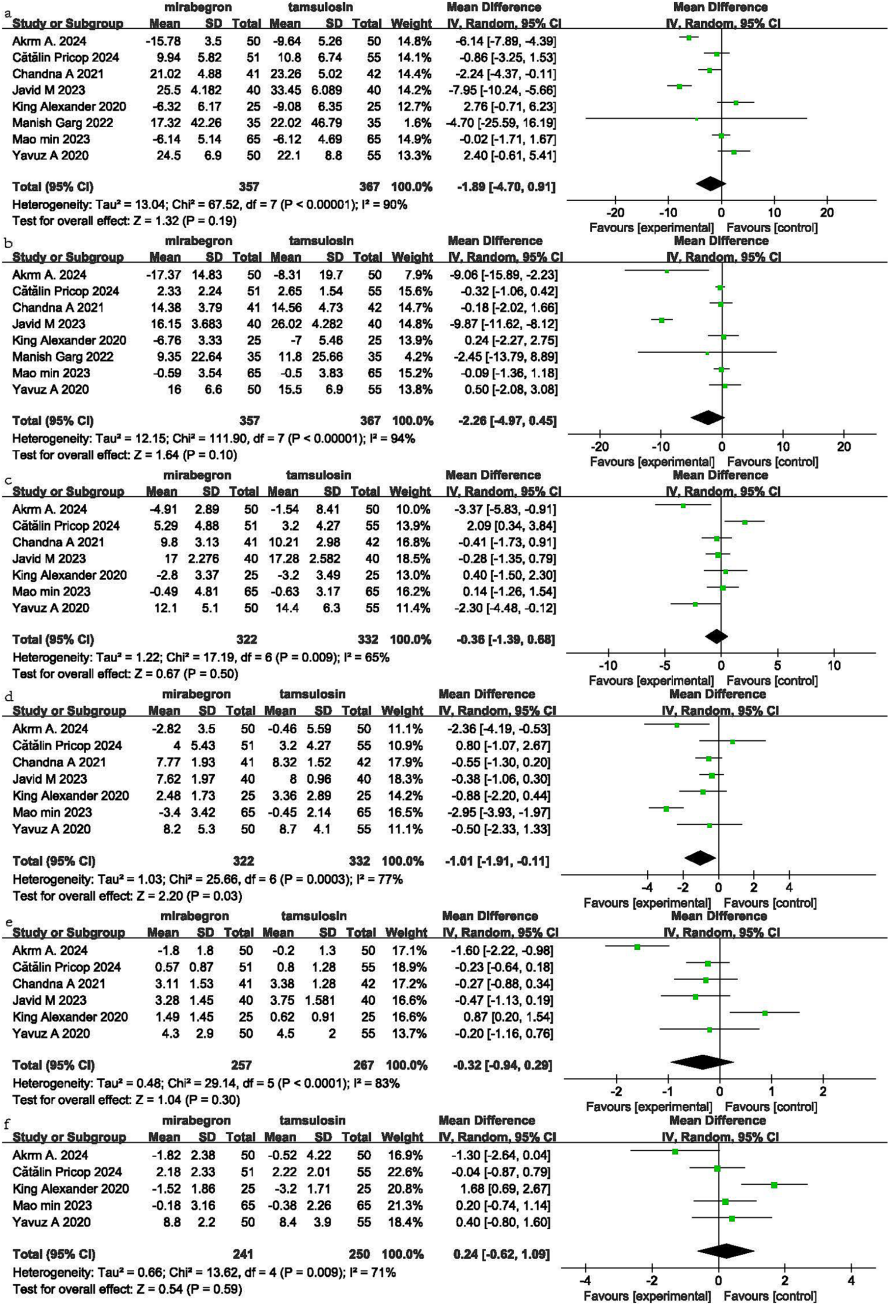
Forest plot of USSQ (**a**: UIS, **b**: PIS, **c**: GHIS, **d**: WPI, **e**: Sexual score, **f**: Additional matters).

**Figure 4:**
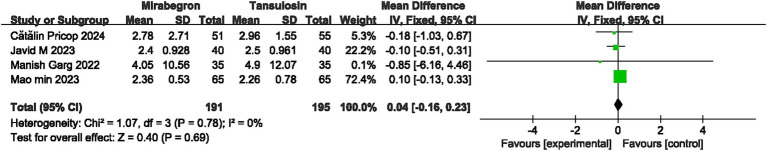
Forest plot of VAS.

Eight RCT studies included USSQ-related data, providing USSQ scores after the administration of both drugs (Figure 3). Based on this, a comparison between mirabegron (M group) and tamsulosin (T group) was made, and the results were as follows: for Urinary index score (UIS) (M group: T group = 357:367), MD = −1.89, 95% CI = −4.70 to 0.91, *p* = 0.19, *I*^2^ = 90%. For Pain index score (PIS) (M group: T group = 357:367), MD = −2.26, 95% CI = −4.97 to 0.45, *p* = 0.10, *I*^2^ = 94%. For General health index score (GHIS) (M group: T group = 322:332), MD = −0.36, 95% CI = −1.39 to 0.68, *p* = 0.50, *I*^2^ = 65%. For Work performance index (WPI) (M group: T group = 322:332), MD = −1.01, 95% CI = −1.91 to −0.11, *p* = 0.03, *I*^2^ = 77%. For Sexual score (M group: T group = 257:267), MD = −0.32, 95% CI = −0.94 to 0.29, *p* = 0.30, *I*^2^ = 83%. For Additional matters (M group: T group = 241:250), MD = 0.24, 95% CI = −0.62 to 1.09, *p* = 0.59, *I*^2^ = 71%. For Visual analogue pain score (VAS) (M group: T group = 191:195): MD = 0.04, 95% CI = −0.16 to 0.23, *p* = 0.69, *I*^2^ = 0% (Figure 4).

### IPSS

**Figure 5:**
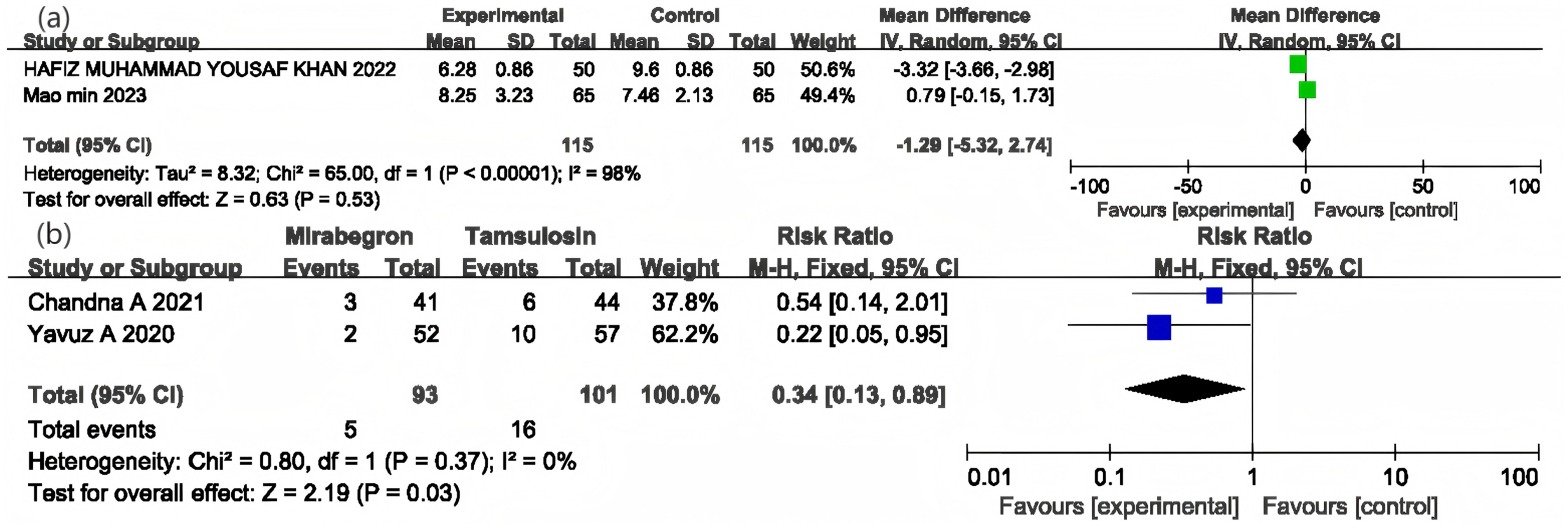
Forest plot of IPSS **(a)** and adverse events after medication **(b)**.

Data regarding IPSS came from two RCTs involving a total of 230 participants (115 in the mirabegron group and 115 in the tamsulosin group). The overall pooled analysis showed no statistically significant difference in improving IPSS scores between the two treatments (MD = −1.29, 95% CI = −5.32 to 2.74, *p* = 0.53, *I*^2^ = 98%) (Figure 5a).

### Safety

Two RCTs, involving a total of 194 participants, were included to evaluate the adverse events associated with medication. The overall analysis demonstrated a significant difference in the occurrence of adverse events between mirabegron and tamsulosin, with a RR of 0.34 (95% CI = 0.13 to 0.89, *p* = 0.03, *I*^2^ = 0%) (Figure 5b).

### Sensitivity analysis

**Figure 6:**
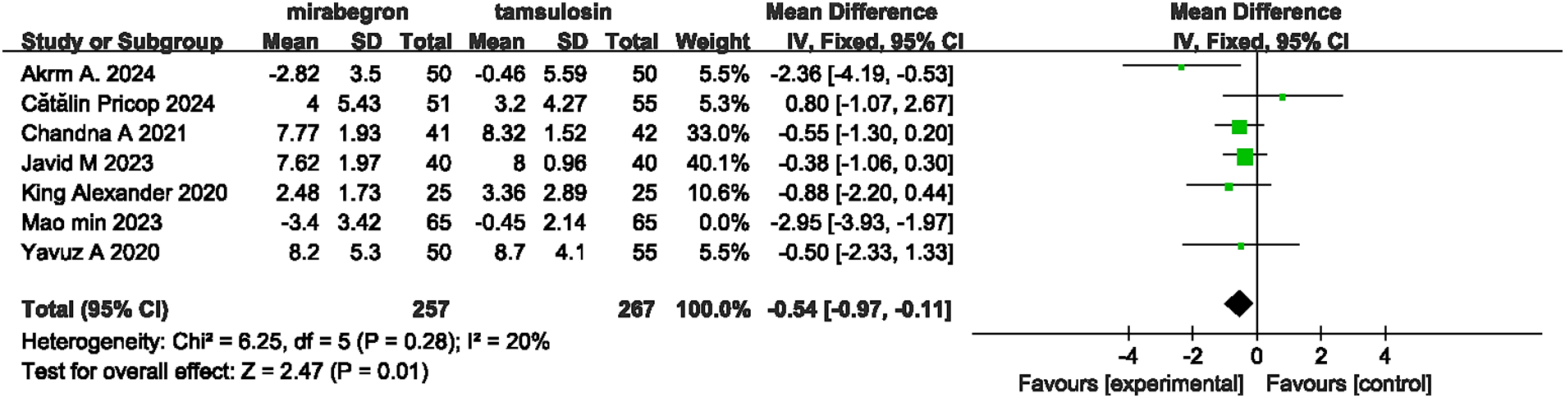
Sensitivity analysis results of WPI.

After excluding one study (34), the WPI result changed from –1.01 (95% CI = −1.91 to –0.11, *p* = 0.03, *I*² = 77%) to –0.54 (95% CI = –0.97 to –0.11, *p* = 0.01, *I*² = 20%) (Figure 6).

## Discussion

Tamsulosin is a potent third-generation α1A adrenergic receptor blocker primarily used to treat benign prostatic hyperplasia and bladder outlet obstruction symptoms. α1A receptors are mainly found in the prostate, ureter, and urethra, with a higher density in the distal ureter and lower density in the bladder (17). Tamsulosin may help prevent symptoms associated with DJ stents by dilating the ureter. Its side effects primarily include orthostatic hypotension and dizziness (17).

Beta-3 adrenergic receptors (β3AR) are expressed in the mucosal and muscular layers of the bladder and ureter, and also in fat, the gastrointestinal tract, and cardiac/vascular tissues (18–20). Some studies suggest that mirabegron inhibits mechanosensitive bladder afferent activity, particularly Aδ fibers, which may be one of the mechanisms for suppressing bladder microactivity (21), after administration of selective β3AR agonists, bladder smooth muscle tissue exhibits a dose-dependent relaxation due to the increase in calcium-regulated potassium channels (22). The most common drug-related AEs are hypertension, nasopharyngitis, and cystitis (23). Some studies have shown that the incidence of hypertension with mirabegron is similar to that of anticholinergic drugs (24, 25). Mirabegron has a lower risk of causing acute urinary retention, and for patients requiring long-term ureteral stent placement due to malignant obstruction, mirabegron has better compliance and persistence compared to other medications (26).

Both mirabegron and tamsulosin are used clinically to manage symptoms related to double-J stents, but their differing mechanisms may lead to variations in efficacy. There are discrepancies between studies regarding the effectiveness of these two medications. Our study summarized and analyzed relevant RCTs from different databases and conducted a meta-analysis based on this data. Most included studies used the USSQ scale, which is very comprehensive (3, 8). It includes assessments of urological symptoms, pain, sexual function, work performance, general health, and other possible issues, allowing for the evaluation of symptoms and quality of life in patients with DJ stents. This scale not only assesses the physical symptoms of patients but also encompasses the psychological, emotional, and social impacts associated with these symptoms, providing a multidimensional reflection of the patient’s struggles post-stent insertion.

We found that mirabegron was better at alleviating WPI, whereas there were no statistically significant differences in urological symptoms, pain, or other symptoms. This suggests that mirabegron and tamsulosin are similar in terms of improving ureteral stent symptoms, while mirabegron may be more effective in improving the daily work performance of patients with DJ stents, which is especially important for many patients.

SRS often lead to a decline in work performance. We believe the reason may be that mirabegron improves bladder storage function. After stone removal surgery, doctors often advise patients to drink more water, but frequent urination reminds them of the potential anxiety caused by the DJ stent inside their body. This can also affect their ability to concentrate on work. Taking mirabegron may help patients return to a healthier work state closer to their pre-surgery condition, making it superior to tamsulosin in terms of work-related symptoms. Additionally, tamsulosin’s side effects, including dizziness and fatigue, may impact daily activities and work performance. Since mirabegron has fewer such side effects, patients may maintain better work performance during treatment.

In terms of adverse events, mirabegron and tamsulosin are generally considered to have a high safety profile, with most side effects being mild to moderate, and severe adverse events being relatively rare (27, 28). However, the risks associated with mirabegron and tamsulosin differ. The former is primarily associated with hypertension, while the latter may lead to hypotension and syncope. Specifically, the incidence rate in the mirabegron group is relatively low (10, 16), with the most commonly reported adverse effects being hypertension, flushing, headache, and fatigue. Long-term use of mirabegron generally results in stable therapeutic effects, with side effects typically being mild. Other long-term complications, apart from hypertension, are rare. However, for patients with pre-existing hypertension or cardiovascular conditions, regular blood pressure monitoring is necessary during long-term use.

Compared to mirabegron, common side effects of tamsulosin include hypotension (particularly orthostatic hypotension) and ejaculatory dysfunction, both of which can affect the patient’s quality of life. Ejaculatory dysfunction occurs only in a subset of patients and is reversible (29, 30). However, it is a significant reason for discontinuation, especially in younger men (31). The mechanism underlying this side effect is related to tamsulosin’s antagonism of the α1-adrenergic receptors, which primarily affects the smooth muscles of the prostate and bladder neck. Additionally, it impacts the muscles involved in ejaculation, leading to issues during ejaculation. If the side effect is pronounced, it can generally be managed effectively by adjusting the dosage (29). Orthostatic hypotension typically presents as dizziness, fatigue, or even syncope upon standing. In patients with pre-existing hypotension or orthostatic hypotension, tamsulosin may exacerbate these symptoms. Among patients with cardiovascular disease, particularly those with heart failure or severe hypotension, tamsulosin may increase the risk of falls and impose additional cardiac burden.

The long-term efficacy and safety of these two medications in managing ureteral-related symptoms have yet to be studied. The studies we included primarily focused on short-term efficacy and safety. No serious adverse effects have been observed with prolonged use of these medications at therapeutic doses (32, 33). While ureteral stents are mainly used in the short term for patients suffering from stone-related obstruction, understanding the long-term efficacy and safety in alleviating SRS is equally important, particularly for patients with malignant ureteral obstruction or benign ureteral strictures who require long-term stent placement.

Two studies reduced the drug dosage by half (13, 34). One study used 0.2 mg of tamsulosin (34), which is half of the commonly used 0.4 mg dose, but this was considered safer. Another study used 25 mg of mirabegron, half the 50 mg dose used in other studies included. Reducing the dose did not show significant differences in efficacy nor increase the incidence of adverse drug reactions, which is why we included these two studies.

Our meta-analysis showed considerable heterogeneity (I^2^ values generally above 70%), indicating that there is high variability among the studies, suggesting that drugs may not be the sole factor contributing to the observed differences. After excluding Mao M’s study, the heterogeneity of WPI significantly decreased, but the combined effect size did not change substantially. This strengthens the credibility of mirabegron’s effect on WPI. After analysis, we believe the heterogeneity may be related to the stent material, as this study indicated in the discussion that it used a new type of ureteral stent. Factors such as the flexibility, diameter, and surface design of different stents may affect patient comfort and exacerbate the occurrence of urinary tract irritation symptoms. Additionally, we believe that the use of antimicrobial drugs postoperatively, the degree of local edema, patient age differences, and individual drug responses may also contribute to postoperative symptom differences. Therefore, although mirabegron has shown consistent efficacy in alleviating WPI, further research is needed to validate its effectiveness. Future studies should standardize stent selection, antimicrobial management strategies, and patient selection criteria, while also collecting more detailed data on clinical characteristics and surgical parameters to accurately identify key factors affecting postoperative symptoms and improve the generalizability of treatment outcomes.

When interpreting the results of this study, several limitations must be considered. First, there is considerable heterogeneity among the included studies. Second, although this analysis included multiple RCTs, some studies had small sample sizes, which may limit the statistical power and prevent the detection of potential small differences. Additionally, the follow-up periods in the included studies were relatively short, as double-J stents are typically removed within 3–4 weeks after surgery. For patients requiring long-term stent placement, the long-term effects of the medication remain to be studied further. The analysis did not separately assess patients with bilateral ureteral stent placement, nor did it account for factors such as the length, thickness, and material of the stents. Only two studies reported adverse drug reactions, suggesting that future RCTs should focus on collecting such data (such as common adverse reactions include dizziness, ejaculation dysfunction, dry mouth, headache, hypertension, constipation, urinary tract infections, and nasopharyngitis) to provide better guidance for clinicians in prescribing medication. Therefore, more large-scale, high-quality RCTs with extended follow-up and comprehensive safety data are needed to provide clearer evidence of the efficacy and safety of these treatments. The mechanisms of action of these two drugs differ significantly, and future basic research can explore the molecular-level differences in their mechanisms, providing a more theoretical basis for clinical drug selection.

## Conclusion

Mirabegron demonstrated better efficacy in terms of the WPI and was associated with fewer side effects overall. Notably, mirabegron may be particularly beneficial for patients who experience tamsulosin-related side effects, such as hypotension or ejaculatory dysfunction. However, there was no significant difference between the two drugs in most aspects of the USSQ or in the IPSS. While these findings suggest that mirabegron could have an advantage in managing DJ stent-related symptoms, it is important to interpret these results with caution due to the study’s limitations, including the small sample size and the short follow-up period. Further research is needed to confirm these findings and identify patient groups who may benefit the most from mirabegron treatment. Specifically, more large-scale, high-quality RCTs with extended follow-up periods and comprehensive safety data are necessary to provide clearer evidence of its efficacy and safety.

## Data Availability

The original contributions presented in the study are included in the article/Supplementary material, further inquiries can be directed to the corresponding author/s.

## Supplementary material

SUPPLEMENTARY TABLE 1 Raw data for meta analysis.

SUPPLEMENTARY FIGURE 1 Funnel plot of the USSQ (**A**: UIS, **B**: PIS, **C**: GHIS, **D**: WPI, **E**: Sexual score, **F**: Additional matters).
