# Supplementary figures and images for: A systematic review and meta-analysis of the efficacy and safety of mirabegron vs. tamsulosin in alleviating double-J catheter related symptoms
Source: Front Med (Lausanne). 2025 Nov 3;12:1669827. doi: 10.3389/fmed.2025.1669827 (PMC12621141; doi:10.3389/fmed.2025.1669827)

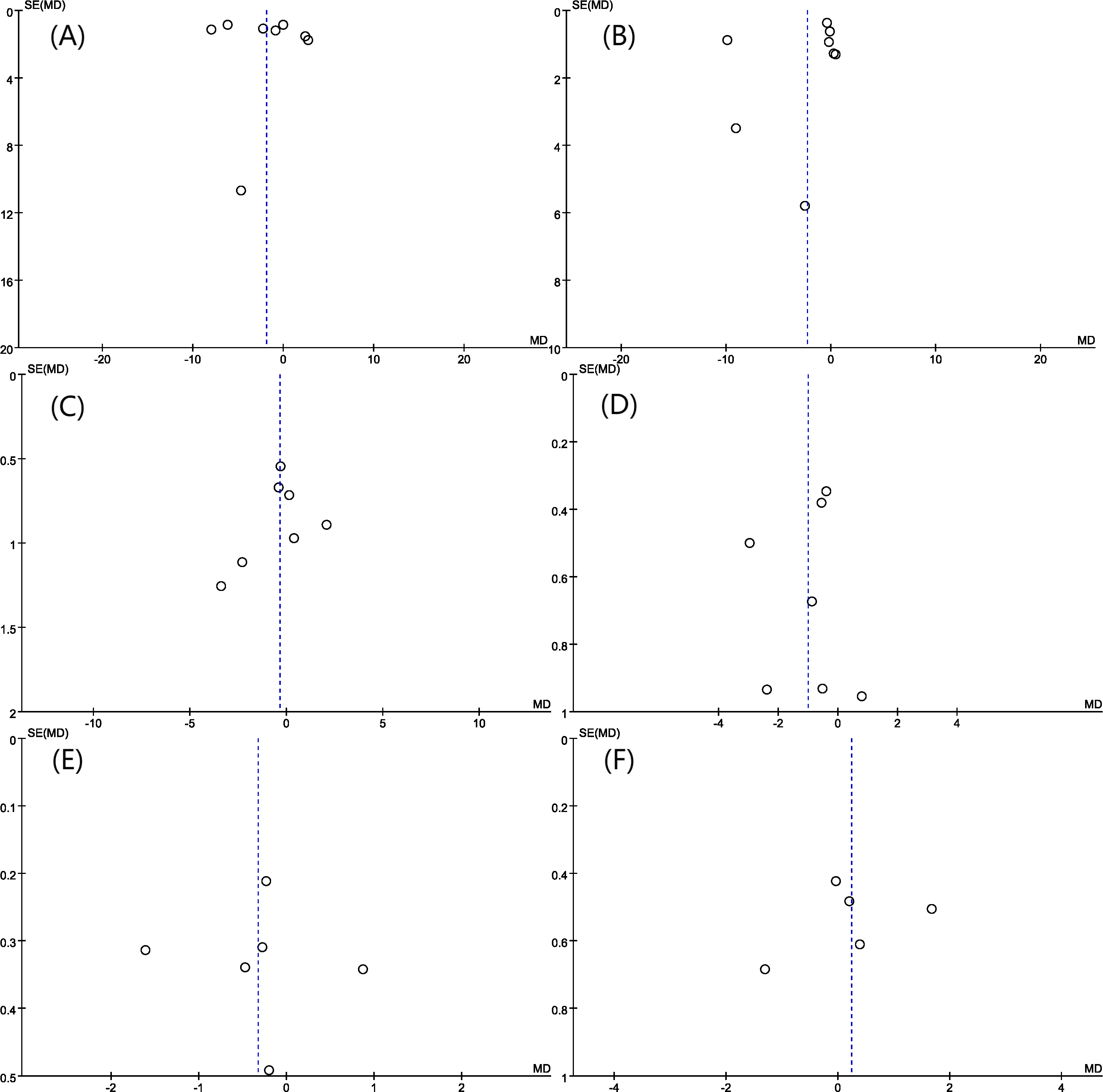

Supplement: SUPPLEMENTARY FIGURE 1 — Funnel plot of the USSQ (A: UIS, B: PIS, C: GHIS, D: WPI, E: Sexual score, F: Additional matters). [file Image_1.PNG]
